# Overview of EWB and Aging

**DOI:** 10.1093/geroni/igab046.783

**Published:** 2021-12-17

**Authors:** Robert Kaplan

**Affiliations:** Stanford University, Stanford University, California, United States

## Abstract

The accumulation of scientific knowledge has been hampered by inconsistent usage of terms and categories. Ontology is the study of categories, their properties, and the relations between them. This presentation considers the definition and measurement of emotional well-being (EWB), a term that has been used inconsistently in research and clinical practice. The category contains eudaimonic and hedonic well-being that represent interrelated but conceptually distinct aspects of mental health. This presentation will review the definition and measurement of EWB and evidence for the validity of the construct. Evidence suggests EWB increases after age 50 and is important for maintenance of cognitive function in old age. Further, low in EWB may be a risk factor for incident ADRD, and is likely to impair cognitive functioning.

